# Dietary Sodium Intake: Knowledge, Attitudes and Practices in Shandong Province, China, 2011

**DOI:** 10.1371/journal.pone.0058973

**Published:** 2013-03-18

**Authors:** Juan Zhang, Ai-qiang Xu, Ji-xiang Ma, Xiao-ming Shi, Xiao-lei Guo, Michael Engelgau, Liu-xia Yan, Yuan Li, Yi-chong Li, Hui-cheng Wang, Zi-long Lu, Ji-yu Zhang, Xiao-feng Liang

**Affiliations:** 1 Division of Non-communicable Disease Control and Community Health, Chinese Center for Disease Control and Prevention, Beijing, China; 2 Shandong Center for Disease Control and Prevention, Jinan, China; 3 National Center for Chronic and Non-communicable Disease Control and Prevention, Chinese Center for Disease Control and Prevention, Beijing, China; 4 Institute of NCD Control and Prevention, Shandong Center for Disease Control and Prevention, Jinan, China; 5 Center for Global Health, Centers for Disease Control and Prevention, Atlanta, Georgia, United States of America,; 6 Chinese Center for Disease Control and Prevention, Beijing, China; Tehran University of Medical Sciences, Iran (Islamic Republic Of)

## Abstract

**Objective:**

To investigate the knowledge, attitudes and practices (KAP) for dietary sodium intake among adult residents of Shandong Province, China

**Methods:**

In 2011, we conducted a cross sectional survey among a representative sample of 15,350 adults aged 18 to 69 years using a standardized questionnaire to assess their KAP for sodium. Variation in the KAPs by gender, and residence location were compared using the Chi-square tests. Predictors for the ‘intention to’ and ‘currently taking action to’ reduce sodium intake were determined by multivariate logistic regression with adjustment for confounding factors.

**Results:**

KAPs for dietary sodium intake among urban residents was generally more favorable than among rural residents. Women were likely to have more favorable KAPs than men. About four fifth of subjects reported that they favored a low sodium diets. However, 31% reported that consumption of less sodium results in less physical strength. Overall, 70% indicated their intention to reduce sodium intake, although only 39 % reported that they had taken action to reduce sodium. Multiple logistic regression analyses indicated that favorable actions to dietary sodium reduction were more likely to occur among those who were aware of the link between sodium and hypertension, and less likely among those who had unfavorable attitudes towards dietary sodium reduction.

**Conclusion:**

Increasing knowledge levels about the benefits of sodium reduction will be a key success factor for effective sodium reduction initiatives and is linked to favorable behavioral change. Emphasis should be placed on the rural area.

## Introduction

The Chinese diet is high in sodium. The National China Nutrition Surveys (1982, 1992, and 2002) and the Chinese Behavioral Risk Factor Surveillance indicated that about 80% of the Chinese exceeded the Chinese Nutritional Society’s (2007) recommended salt (sodium chloride) intake upper level of 6 gram/day. In 2010, the estimated average salt intake of Chinese citizens was 9.1 grams/day in urban areas, and 11.5 grams/day in rural areas. [1]

Globally, a causal link between sodium intake and high blood pressure levels is well-established. [2]–[3] In China, a considerable body of literature also links higher sodium intake with higher blood pressure. Excess dietary sodium is a major contributor to hypertension. [4] From 1959 to 2002, the prevalence of hypertension in China increased by more than three-fold, from 5.1% to 18.0%. And, making matters worse, in 2002, only 6% of these hypertensive individuals had their blood pressure under control. [5] In China, hypertension is a major contributor to cardiovascular disease, which accounts for 40% of the deaths, [6] and 23% of the health care costs. [7]

Evidence suggests that modest reductions in dietary sodium could substantially reduce cardiovascular events and medical costs, and should be a public health priority. [8] Internationally, calls have been made for dietary sodium reduction to be a major intervention for prevention and control of non-communicable diseases. [9] Many countries, including Japan, the United Kingdom, Finland, Portugal, and the United States, have reduced population-wide sodium intake through a combination of regulations on the sodium content in processed foods, labeling of processed and prepared foods, public education, and collaboration with the food industry. [10]–[12] In China, a major challenge to sodium intake reduction effort, in contrast to western countries, is that majority of sodium comes from home cooking, not commercially processed foods. A recent study suggested that most dietary sodium (76%) in China was from salt added in home cooking. [13] In contrast, processed foods contributed heavily to sodium intake in the UK (95%), and the US (71%). [13] This study also reported that dietary sodium was about 50% less in southern China than the northern China. [13] To prevent and control hypertension and improve health, efforts to remove excess sodium from diets in China should focus on reducing sodium in home cooking.

Shandong Province is one of the most populous provinces in China. Sodium intake by most Shandong residents considerably exceeds the recommended limit. In 2002, the average daily salt intake was 12.6 gram, and the prevalence of hypertension was 25.1%. A cross-sectional study conducted in rural Shandong province found that 43.8% of the population had hypertension. Among those with hypertension, only 26.2% were aware of their hypertension, 22.2% were currently undergoing antihypertensive treatment, and only 3.9% had their blood pressure controlled. [14] To reduce sodium in the Chinese diet, in 2011, the Chinese Ministry of Health (MoH) selected Shandong province as a national pilot area for sodium reduction and the Shandong MOH Action on Salt and Hypertension (SMASH) Project was formed. In order to help develop effective interventions for the SMASH Project, in 2011, a base-line survey was conducted to assess the sodium intake KAP, salt consumption, and the prevalence of hypertension among the Shandong residents. This study analyzes this baseline survey to investigates the current knowledge, attitudes, and practices related to sodium and hypertension among the general adult Shandong population (aged 18–69) and to inform effective sodium reduction initiatives.

## Materials and Methods

During June to July, 2011, a cross-sectional survey was conducted in Shandong province. Multi-stage (4-stage) cluster sampling was used to select a provincially representative sample of the adult population (aged 18–69). Briefly, a total of 140 counties/districts in Shandong province were stratified by geographic distribution (Eastern, Central Southern, and North Western), and by residence (urban versus rural). Twenty counties/districts were selected. Using a proportional probability sampling (PPS) method, three townships (in rural areas) or two streets (in urban areas) were selected from each selected county or district. Also using PPS, 3 villages/neighborhoods were selected from each sampled township/street. In every selected village/neighborhood, 100 subjects were selected by random sampling. A total of 15,600 subjects were sampled.

A face to face, close-ended questionnaire was administered by trained public health professionals, all of whom had previous experience in conducting interviews in similar health surveys. Questions collected information on: demographics (gender, age, ethnicity, education, profession, health insurance information), health status (hypertension, diabetes), lifestyle (smoking, alcohol use, physical activity, and diet), and knowledge, attitudes and practices related to sodium and hypertension (relationship between sodium and hypertension, consequences of hypertension, perception of salt consumption, self-reported consumption, intention to reduce salt consumption, and practices towards reducing sodium consumption).

Physical measurements taken in all subjects included: height, weight, waist circumference, and blood pressure. Of 15,600 subjects, 15,350 subjects with complete data were included for this analysis (response rate 98.4%). The survey received ethical approval from the Ethics Committee of the Shandong Center for Disease Control and prevention, and participants provided written informed consent to participate in this study.

Educational level and annual household income were used as indicators for social economic status (SES). We divided participants into five groups by education level: no education, primary school, junior high school, senior high school, or any college and above. Annual household income level was classified in high, middle, and low, according to tertiles.

Participants were designated as hypertensive if their systolic BP was ≥140mmHg and/or diastolic BP≥90mmHg and/or they were currently taking antihypertensive medication.

### Statistical analysis

Statistical analyses were performed with SPSS 17.0 for windows (Chicago, IL). Survey weight (total weight = design weight * post-stratification weight) was applied to calculate weighted proportions. Design weight was calculated to account for different factors, including cluster design, strata, and individual. The population data of Shandong province was used to generate post-stratification weight. Difference in proportions of KAP rates were compared using the Chi-square test. The significance level was set at p≤0.05. Logistic regression was applied to examine the relationship between indicators of socioeconomic status, knowledge and attitudes related to sodium and hypertension, and intention and practice towards sodium reduction, from which odds ratios and 95% confidence intervals (95%CI) were computed. Tests for trends were calculated in the same logistic regression model using educational level and annual household income as continuous variables. The models were adjusted for potential confounders which included age, sex, marital status, residence, region, and hypertension status.

## Results

### Study subjects

Out of 15,350 subjects, there were 7,683(50%) males and 7,667(50%) females, of which 4,798 (31%) resided in urban areas and 10,524 (69%) in rural areas. A greater proportion of subjects reported lower educational levels and household income levels in rural areas than in urban areas. (Table 1)

**Table 1:** Socioeconomic Status by Demographics Characteristics and Hypertension Status.

	Total	Education level					Household income		
		No education	Primary school	Junior high school	Senior high school	Any college	Low	Middle	High
Total	15350	2108(13.8)	3022(19.7)	6624(43.2)	2372(15.5)	1196(7.8)	5262(34.7)	4859(32.0)	5057(33.3)
**Age**									
18∼	**3861**	93(4.4)	257(8.5)	1879(28.4)	920(38.8)	712(59.5)	985(18.7)	1298(26.7)	1540(30.5)
30∼	**3808**	211(10.0)	729(24.1)	2057(31.1)	509(21.5)	302(25.3)	1155(21.9)	1243(25.6)	1374(27.2)
40∼	**3151**	279(13.2)	687(22.7)	1611(24.3)	460(19.4)	114(9.5)	1024(19.5)	1056(21.7)	1042(20.6)
50∼	**2215**	632(30.0)	512(16.9)	673(10.2)	362(15.3)	36(3.0)	915(17.4)	673(13.9)	606(12.0)
60–69-year-old	**2287**	893(42.4)	837(27.7)	404(6.1)	121(5.1)	32(2.7)	1183(22.5)	589(12.1)	495(9.8)
**Sex**									
Male	**7672(50.1)**	544(25.8)	1385(45.8)	3659(55.2)	1405(59.2)	679(56.8)	2522(48.5)	2428(50.0)	2621(51.8)
Female	**7650(49.9)**	1564(74.2)	1637(54.2)	2965(44.8)	967(40.8)	517(43.2)	2710(51.5)	2431(50.0)	2436(48.2)
**Marital status**									
Yes	**13312(86.9)**	1907(90.5)	2817(93.3)	6065(91.6)	1822(76.8)	701(58.6)	4633(88.1)	4301(88.5)	4253(84.1)
No	**2007(13.1)**	201(9.5)	203(6.7)	559(8.4)	549(23.2)	495(41.4)	627(11.9)	558(11.5)	802(15.9)
**Urban/rural**									
Urban	**4798(31.3)**	399(18.9)	695(23.0)	1741(26.3)	1099(46.3)	864(72.2)	893(17.0)	1437(29.6)	2462(48.7)
Rural	**10524(68.7)**	1709(81.1)	2327(77.0)	4883(73.7)	1273(53.7)	332(27.8)	4369(83.0)	3422(70.4)	2595(51.3)
**Geographic region**									
Central-south	**5930(38.7)**	846(40.1)	1007(33.3)	2357(35.6)	1039(43.8)	681(56.9)	1968(37.4)	1898(39.1)	2052(40.6)
West-north	**5691(37.1)**	1068(50.7)	1323(43.8)	2486(37.5)	636(26.8)	178(14.9)	2105(40.0)	1896(39.0)	1562(30.9)
East	**3701(24.2)**	194(9.2)	692(22.9)	1781(26.9)	697(29.4)	337(28.2)	1189(22.6)	1065(21.9)	1443(28.5)
**Hypertension**									
Yes	**3768(24.6)**	842(40.0)	920(30.5)	1360(20.5)	500(21.1)	146(12.2)	1487(28.3)	1168(24.1)	1071(21.2)
No	**11542(75.4)**	1265(60.0)	2098(69.5)	5259(79.5)	1871(78.9)	1049(87.8)	3772(71.7)	3686(75.9)	3982(78.8)

Adjusted for: residence (urban vs. rural), education, family income, hypertension status , sex, regional location, and marital status

### Knowledge on sodium and hypertension

The rates of KAP were generally more favorable among urban residents than their rural counterparts.(Table 2). About half of the population were aware of the relationship of sodium with hypertension, with females slightly more likely to be aware of the relationship of sodium with hypertension, than males (P<0.05). However, less than one third of subjects knew the Chinese Nutrition Guideline recommendation of daily salt intake of less than 6 grams.

**Table 2:** Knowledge, attitudes, and practices for Dietary Sodium among Shandong Residents: n (%).

	Urban				Rural				
	Total	Men	Women	P*	Total	Men	Women	P*	P^#^
**Knowledge**									
Threshold of hypertension	1983(39.9)	1012(42.0)	971(37.9)	<0.001	2892(27.0%)	1563(29.2)	1329(24.7)	<0.001	<0.001
Stroke is one of the consequences of hypertension	2751(56.6)	1335(55.7)	1416(57.5)	0.05	4631(42.7)	2319(43.1)	2312(42.3)	0.87	<0.001
Heart disease is one of the consequences of hypertension	2753(51.6)	1267(51.6)	1306(51.5)	0.43	3655(33.7)	1892(35.1)	1763(32.3)	0.02	<0.001
Recommended limit of salt by the Chinese Nutrition Guideline	1466(29.3)	717(29.6)	751(28.9)	0.39	2049(19.2)	1062(20.0)	987(18.3)	0.10	<0.001
As salt intake decreases, so does blood pressure	2899(56.7)	1391(54.7)	1508(58.6)	0.002	5265(50.0)	2545(48.7)	2703(51.3)	0.003	<0.001
As salt intake increases, so does blood pressure	3077(60.3)	1484(58.3)	1593(62.3)	0.004	5078(49.0)	2497(48.1)	2581(49.9)	0.03	<0.001
**Attitude**									
Consumption of less salt results in less physical strength	1366(30.8)	708(31.0)	658(30.5)	0.07	3270(31.6)	1690(32.4)	1580(30.7)	0.05	<0.001
Not necessary to reduce sodium intake, unless one is sick	835(19.6)	430(19.6)	405(19.)	0.27	1614(16.4)	846(17.2)	768(15.6)	0.06	0.008
Approval of low sodium diet	4246(87.2)	2048(84.2)	2198(90.2)	<0.001	9561(90.3)	4726(89.0)	4835(91.7)	<0.001	<0.001
Food package should claim food labels	3831(78.6)	1910(78.5)	1921(78.7)	0.95	8328(77.9)	4268(80.0)	4060(75.8)	<0.001	0.49
Food labeling of sodium contents help choose low-sodium diet	3611(73.9)	1782(73.7)	1829(74.1)	0.58	7737(73.1)	3916(74.0)	3821(72.1)	0.02	<0.001
**Practices**									
Self-reported overconsumption of salt	1284(28.1)	662(26.2)	800(32.2)	<0.001	2897(27.1)	1604(29.9)	1965(37.0)	<0.001	<0.001
Intention to reduce dietary salt intake	4094(85.2)	1964(81.7)	2130(88.7)	<0.001	9131(86.1)	4461(83.8)	4670(88.3)	<0.001	<0.001
Have taken action to reduce dietary sodium intake	2213(45.6)	910(37.5)	1303(53.7)	<0.001	3658(34.8)	1581(30.3)	2077(39.4)	<0.001	<0.001
Have used salt spoon	745(14.1)	332(13.0)	413(15.2)	0.002	391(3.8)	211(4.1)	180(3.5)	0.30	<0.001
Have read food labels of sodium contents	694(13.9)	310(12.7)	384(15.1)	0.01	968(9.7)	492(9.8)	476(9.6)	0.90	<0.001

* : significant difference between females and males in the KAP rates

#: significant difference between urban and rural in the KAP rates

### Attitude towards sodium reduction

Most participants (80%) indicated they favored low sodium diets (Table 2). Regardless of residence (urban vs. rural), compared to males, females were more likely to report favoring a low sodium diets (P<0.001). Nevertheless, 31% of subjects (urban 31%; rural 32%) reported that less sodium consumption resulted in less physical strength.

### Practices towards sodium reduction

Approximately one third of participants (urban: 27%; rural: 28%) reported that they consumed excess amounts of salt; Over four fifth of subjects (urban: 85%;rural: 86%) indicated their intention to reduce sodium intake. Among subjects who reported no intention to reduce sodium, 80% were afraid that less salt affected food taste, followed by 9.9% who were afraid that less salt intake resulted in less physical strength.

However, less than half of the subjects (urban: 46%;rural: 35%) reported that they had already taken action towards sodium reduction. Females were more likely to perceive themselves at risk of consuming excess sodium, to report an intention to reduce sodium intake, and to report that they had already taken action towards sodium reduction (P<0.05).

Regarding actions to reduce sodium intake, most subjects (urban: 96%, rural: 96%) reported using less salt when cooking, followed by using less pickles (urban: 54%%, rural: 45%), and adding salt later or using table salt (urban: 28%, rural: 20%) (Table 3). Many subjects, in particular those in urban areas, reported using green onion or garlic to improve the taste of food when not using salt (20%). Some subjects also used non -sodium condiments such as vinegar (15%).

**Table 3:** Measures Used to Control Dietary Sodium Intake (%).

	Urban	Rural	P* value
Use less salt when cooking	96.2	95.7	0.22
Add salt later or use table salt instead	27.6	19.7	<0.001
Use less high sodium condiments	24.9	12.2	<0.001
Use less pickles	54.0	44.6	<0.001
Use low sodium processed food	21.4	10.4	<0.001
Use vinegar when cooking	15.1	5.0	<0.001
Use green onion, garlic to improve the taste	20.2	8.7	<0.001

* significant difference between urban and rural

Multiple logistic regression analysis, adjusting for age, gender, marital status, residence, region, and hypertension status, found that, compared to persons with no education, persons with higher education level were more likely to report they had already taken action towards sodium reduction (urban: OR =  2.15, 95% CI 1.67–2.77; rural: OR = 2.20, 95% CI 1.91–2.54, see Table 4). Persons with high household incomes were also more likely to report that they have taken action towards sodium reduction (urban: OR = 1.29, 95% CI 1.08–1.54; rural: OR = 1.20, 95% CI 1.09–1.34).

**Table 4:** Association (crude and adjusted odds ratio and 95%CI) between Socioeconomic Status and Intention and Action to Reduce Dietary Sodium Intake by Urban and Rural Status.

	Intention to reduce dietary salt intake				Have taken actions to reduce dietary salt intake			
	Urban		Rural		Urban		Rural	
	Crude OR	Adjusted OR	Crude OR	Adjusted OR	Crude OR	Adjusted OR	Crude OR	Adjusted OR
**Education level**								
No education	1	1	1	1	1		1	1
Primary school	1.18(0.86–1.62)	1.54(1.11–2.13)	1.29(1.08–1.53)	1.54(1.29–1.84)	1.04(0.80–1.34)	1.43(1.09–1.87)	1.18(1.03–1.36)	1.56(1.35–1.80)
Junior high school	1.41(1.07–1.86)	2.31(1.69–3.15)	1.51(1.30–1.76)	2.03(1.69–2.43)	1.15(0.92–1.45)	2.15(1.67–2.77)	1.39(1.23–1.56)	2.20(1.91–2.54)
Senior high school	1.68(1.24–2.28)	2.76(1.97–3.86)	1.83(1.47–2.28)	2.60(2.03–3.32)	1.70(1.34–2.16)	3.39(2.60–4.43)	1.54(1.32–1.80)	2.66(2.23–3.18)
Any college	1.81(1.31–2.49)	3.33(2.31–4.81)	1.93(1.32–2.82)	2.80(1.87–4.20)	1.63(1.27–2.08)	2.18(1.93–2.47)	1.76(1.38–2.24)	3.21(2.46–4.21)
P for trend	<0.001	<0.001	<0.001	<0.001	<0.001	<0.001	<0.001	<0.001
**Income**								
High	1.34(1.09–1.65)	1.44(1.16–1.78)	1.30(1.13–1.51)	1.31(1.12–1.52)	1.57(1.34–1.84)	1.29(1.08–1.54)	1.14(1.03–1.27)	1.20(1.09–1.34)
Middle	1.10(0.88–1.38)	1.15(0.91–1.44)	1.12(0.98–1.27)	1.10(0.96–1.26)	1.23(1.03–1.46)	1.73(1.47–2.04)	1.04(0.95–1.15)	1.06(0.96–1.17)
Low	1	1	1	1	1	1	1	1
P for trend	0.003	<0.001	<0.001	0.001	<0.001	<0.001	0.013	0.001

Note: Adjusted for potential cofounders which included age, sex, marital status, residence, region, and hypertension status.

Additional multiple logistic regression analyses which controlled for key confounders, found practices towards sodium reduction were more likely to be taken by those who were aware that sodium intake was associated with increased blood pressure, compared to those who were not aware (:OR = 2.17, 95% CI 2.01–2.34); by those who perceived themselves at risk of consuming excess sodium (OR = 2.00, 95% CI 1.84–2.17); and by those who reported intention to reduce sodium intake (OR = 4.06, 95% CI 3.56–4.63) (Table 5). However, practices towards sodium reduction were less likely to be taken by those who had unfavorable attitudes towards sodium reduction, for example, subjects who felt that reduced sodium consumption results in less physical strength (OR = 0.87, 95% CI 0.81–0.94), and subjects who considered low salt levels in the diet reduces taste (OR =  0.36, 95% CI 0.32–0.40).

**Table 5:** Association between knowledge, perceived risk, and barriers, with the intention to reduce salt intake, and on taking action to reduce salt intake.

	Intention to reduce salt intake		Take action to reduce salt intake	
	Crude OR	Adjusted OR	Crude OR	Adjusted OR
Knowing the diagnostic standards of hypertension				
Yes	1.71(1.54–1.91)	1.58(1.40–1.76)	1.88(1.75–2.01)	1.63(1.51–1.76)
No				
Knowing the limit of salt				
Yes	1.72(1.52–1.95)	1.68(1.52–1.85)	2.38(2.21–2.58)	2.12(1.95–2.31)
No				
Know that less salt help reduce blood pressure				
Yes	2.50(2.27–2.76)	1.59(1.44–1.76)	2.78(2.59–2.98)	2.44(2.27–2.62)
No				
Know that excess salt increase blood pressure				
Yes	2.14(1.95–2.34)	1.62(1.47–1.79)	2.49(2.32–2.67)	2.17(2.01–2.34)
No				
Self-report excess consumption of salt				
Yes	1.19(1.08–1.32)	1.002(1.000–1.003)	2.01(1.87–2.18)	2.00(1.84–2.17)
No				
Less salt consumption results in less physical strength				
Yes	0.81(0.73–0.89)	0.84(0.76–0.92)	0.82(0.77–0.88)	0.87(0.81–0.94)
No				
Not necessary to reduce salt consumption				
Yes	0.80(0.71–0.90)	0.84(0.74–0.94)	0.95(0.87–1.04)	0.97(0.88–1.07)
No				
Low sodium diet affect taste				
Yes	0.29(0.26–0.32)	0.29(0.26–0.33)	0.36(0.32–0.40)	0.36(0.32–0.40)
No				
Have used salt spoon				
Yes	1.14(1.18–1.76)	1.26(1.02–1.54)	3.96(3.47–4.52)	3.10(2.70–3.56)
No				
Have read health claims				
Yes	1.75(1.46–2.09)	1.54(1.29–1.85)	3.38(3.03–3.76)	3.03(2.70–3.39)
No				
Know salt substitute				
Yes	1.29(1.12–1.50)	1.11(0.96–1.30)	2.56(2.33–2.82)	2.14(1.93–2.37)
No				
Intention to reduce salt				
Yes	–	–	4.26(3.74–4.84)	4.06(3.56–4.63)
No	–	–		

## Discussion

Excess dietary sodium is a major contributor to hypertension and a critical public health issue in China. In 2011, the estimated average intake of sodium among adults (18 to 69 years old) in Shandong province was 12.5 gram/day. Lower than 10% of residents were below the recommended maximum intake of 6 gram/day (unpublished data). However, only one third of the subjects perceived themselves as consuming excess amounts of sodium. Nevertheless, sodium reduction in the diet was practiced by 52.7% of the population in the urban areas and 38.3% in rural areas. Findings from the other component of SMASH base-line survey indicated that 81% of the sodium comes from condiments, including salt, soy source and monosodium glutamate (MSG).

A simplistic approach for reducing sodium reduction is to increase population-level awareness that most are getting too much sodium, then expect changed behaviors that result in lower sodium intake. However, changing dietary sodium intake in a population that has adapted to a high sodium diet is not easy and requires a number of complementary strategies. This study has provided important information on the current state of knowledge, attitudes, and practices related to sodium and hypertension among residents in Shandong province. In the present study, we found good levels of knowledge and a favorable attitudes towards sodium reduction. Residents in Shandong province seem prepared to accept the importance of reducing sodium intake. Our findings provide evidence that will enable the development of effective public education initiatives. Such initiatives may aim to educate the public on the relationship between sodium and health, to increase the public’s demand for lower sodium diets, and promote individual dietary change.

The Health Belief Model postulates that when an individual perceives a threat from a disease and perceived benefits from preventive actions exceed the barriers then the individual is likely to take preventive action. [15] Our study revealed that Shandong residents had a good knowledge about the links between sodium and hypertension, and those who were aware of the links, were more likely to take action towards sodium reduction. However, they still lacked specific knowledge on the 2007 recommendations concerning the maximum intake of salt at 6 gram/day. [16] This may partly explain why many residents did not think their sodium intake was a problem. Additionally, our study suggests that those who perceived themselves at risk of consuming excess sodium were more likely to take action towards sodium reduction. Furthermore, previous findings suggested that people may have resistance to adopting reduced-sodium diets because they are largely asymptomatic, and may fail to perceive a benefit from a diet that they consider too restrictive. [17], [18] In fact, sodium reduction is beneficial in both normotensive and hypertensive people. A review of randomized controlled trials which aimed at reducing dietary sodium with follow-up of 6 months or longer, found that systolic and diastolic blood pressure were reduced by an average of 1 mmHg in normotensives and by an average of 2–4 mmHg in hypertensives. [19] Hence, the recommended limit of salt intake, as well as the relationship of sodium to hypertension should be key messages in public education initiatives to help the public understand problems of excess consumption of sodium. In addition, it is important to highlight that reduced sodium intake is beneficial for both hypertensive and normotensive people, although it affects hypertensive people to a greater degree.

The majority of Shandong residents believed that low sodium diets have less taste, and many residents reported low sodium intake reduces physical strength. Our study suggests that persons who had unfavorable attitudes towards sodium reduction, were less likely to take action towards sodium reduction. Therefore, public education initiatives should also focus on these perceived barriers of taste and lost physical strength. Preliminary results from this study suggested that survey participants who were older were more likely to report that less sodium intake results in less physical strength. The Chinese Nutrition Guidelines make recommendations that Chinese people should avoid consuming too much sodium. Effective public education should also inform people that cooking with less sodium without affecting physical strength.

The Social Cognitive Theory suggests that people need mastery of both knowledge and skills to perform a given behavior. [15] It has been suggested that spreading information in an education campaign is definitely a requirement for dietary sodium reduction, but equipping population with the skills to make the necessary changes is equally important. [20] Therefore, effective public education initiative should not only disseminate information on the links between sodium and health, but also provide practical and culturally appropriate means to change their diet. First, public education initiatives should promote less use of “hidden sodium”. Soy sauce, monosodium glutamate (MSG), and pickles, are major dietary sources of sodium in China. For example, regular soy sauce contains 1.5 grams sodium/10 milliliter. The per capita consumption of soy sauce in china is 10 milliliter per day. [7] Hence, soy sauce consumption already provides 25% of the recommended maximum intake. Awareness campaigns should aim to increase people’s awareness on this “hidden sodium”, and help people understand that there are other sources of sodium in the diet, in addition to salt. Second, the use of low sodium condiments, or alternative forms of flavoring should be recommended. Our study found that the most frequently reported practice to reduce sodium intake was reducing sodium while cooking, while relatively few reported increased use of non-sodium containing condiments, such as vinegar, green onions, and garlic. Third, the ‘salt spoon’ has been proven to be an effective strategy to help people make the change towards sodium reduction. [21] We found the ‘salt spoon’ was acceptable in Shandong province, in particular in the urban areas, although about one third of those who used a salt spoon did not know how to use it correctly. In terms of the source of salt spoons, 26–38% of them were distributed by health professionals, and 30–45% was purchased by individuals themselves. Therefore, public education initiatives should focus on training the public how to use ‘salt spoons’ in addition to distributing them.

With urbanization and globalization in China, there are signs of a gradual transition from traditional diets to consumption of more imported foods, processed foods and eating meals outside the home. The base-line survey reported that 32.7% of residents (40.0% of urban and 29.8% of rural residents) ate meals outside the home. Processed food and restaurant food often contain higher sodium contents for either palatability or food safety reasons, and thus the trend is likely to contribute to high sodium consumption.

Food labeling has been shown to be effective in reducing sodium intake in developed countries. [10] China started implementing voluntary food labeling in 2007. This program requires manufactures optionally to provide sodium in the list of nutrients on packaged food labels. We found that most Shandong province residents supported food labeling, and believed food labeling would help them follow a low sodium diet However, very few residents reported reading food labels while purchasing. Public education initiatives should encourage manufactures to provide sodium content information on packaged food labels, and educate the public how to read food labels to enable them to make healthy food choices.

Women, gatekeepers to food and health in the household, are an important group to target with the educational initiative. In the UK, in 2003, the Food Standards Agency (FSA) coordinated a public awareness campaign which mainly targeted adult females. This campaign successfully educated consumers on the relationship between sodium and health, increased consumer’s demand for low-sodium products, and educated consumers on making healthy food choices. [22] We found dietary sodium intake KAPs of urban residents were generally better than for those living in rural areas. Hence, special attention should be given to all residents, and especially women, in rural areas. Rural residents often were not well educated indicating that awareness campaigns should aim to develop messages that can be easily understood by the rural residents with lower education levels.

Limitation of this study includes self-reported attitude and practices of participants for having low or high sodium diet, which may overestimate or underestimate their actual attitude and practices related to sodium consumption. However, findings presented in this study demonstrate that most residents in Shandong province will accept dietary sodium reductions. Future effective public education initiative should highlight the benefits of sodium reduction, with a focus of perceived barriers of physical strength and tastes, but also provide practice and culturally appropriate meals to change their diet. The public education and awareness efforts should target all women and should also focus on rural populations.
